# Integrated transcriptomic and TCR profiling reveals local immune dysregulation of T cells in Gl aGvHD

**DOI:** 10.1038/s41409-026-02942-w

**Published:** 2026-06-25

**Authors:** Manisha Goel, Anne Eugster, Susanne Reinhardt, Ulrike Friedrich, Andreas Dahl, Linda Wolters, Luise Lieth, Katja Steinberg-Bains, Marc Schmitz, Tilman D. Rachner, Anna R. Poetsch, Desiree Kunadt, Raphael Teipel, Jan Moritz Middeke, Katja Sockel, Friedrich Stölzel, Malte von Bonin, Johannes Schetelig, Andreas Hartig, Martin Bornhäuser, Ezio Bonifacio, Cornelia S. Link-Rachner

**Affiliations:** 1 Center for Regenerative Therapies Dresden, Faculty of Medicine, Technische Universität Dresden, Dresden, Germany; 2 Dresden-concept Genome Center (DcGC), Center for Molecular and Cellular Bioengineering (CMCB) Technology Platform, Technische Universität Dresden, Dresden, Germany; 3 German Center for Diabetes Research (DZD e.V.), Neuherberg, Germany; 4 Paul Langerhans Institute Dresden of the Helmholtz Center Munich, Faculty of Medicine and Universitätsklinikum Carl Gustav Carus, Technische Universität Dresden, Dresden, Germany; 5 Medizinische Klinik und Poliklinik I, Universitätsklinikum Carl Gustav Carus, Technische Universität Dresden, Dresden, Germany; 6 Institute of Immunology, Medical Faculty Carl Gustav Carus, Technische Universität Dresden, Dresden, Germany; 7 National Center for Tumor Diseases (NCT/UCC) Dresden, a partnership between DKFZ, Faculty of Medicine and Universitätsklinikum Carl Gustav Carus, Technische Universität Dresden, and Helmholtz-Zentrum Dresden-Rossendorf (HZDR), Dresden, Germany; 8 German Cancer Consortium (DKTK), Partner Site Dresden, and German Cancer Research Center (DKFZ), Heidelberg, Germany; 9 Medizinische Klinik und Poliklinik III, Universitätsklinikum Carl Gustav Carus, Technische Universität Dresden, Dresden, Germany; 10 Biomedical Genomics, Biotechnology Center, Center for Molecular and Cellular Bioengineering, Technische Universität Dresden, Dresden, Germany; 11 Division of Stem Cell Transplantation and Cellular Immunotherapies, University Hospital Schleswig-Holstein, Kiel, Kiel University, Kiel, Germany; 12 Institut für Pathologie, Universitätsklinikum Carl Gustav Carus, Technische Universität Dresden, Dresden, Germany

**Keywords:** Graft-versus-host disease, T cells, Graft-versus-host disease, Allotransplantation, T-cell receptor

Allogeneic hematopoietic cell transplantation (allo-HCT) is an effective therapy for hematological malignancies but is limited by acute graft-versus-host disease (aGvHD), particularly in the gastrointestinal (GI) tract. Diagnosis of gastrointestinal aGvHD (GI aGvHD) still relies on histopathology, and ~30% of patients develop steroid-refractory disease with poor prognosis and unclear mechanisms [1].

Donor T cells are key mediators of GvHD, and T cell receptor (TCR) repertoire profiling can reveal immune dysregulation through analysis of clonal expansion, diversity, and specificity [2, 3]. Studies in blood have reported variable TCR diversity depending on disease context, while tissue-based analyses suggest reduced diversity with private clonal expansions not reliably detected in circulation [2].

Identification of GvHD-specific TCR clonotypes remains a major challenge, as no shared clonotypes or clear antigen specificities have been established. Emerging computational approaches may enable inference of antigen specificity but have not yet been widely applied in GvHD [4]. This is particularly relevant for steroid-refractory disease, where persistent tissue-resident clonotypes may drive pathology [5].

While TCR sequencing provides insights into clonality, transcriptomic profiling reveals T cell function and regulation. However, integrated analyses combining both approaches in GvHD are limited.

In this study, we analyzed paired gastrointestinal biopsies and peripheral blood samples collected at the diagnosis of GI aGvHD. Using an integrated workflow of flow cytometry, gene expression profiling, and TCR sequencing, we investigated T cell characteristics in both tissue and blood.

## Methods

Patients were prospectively recruited between 2021 and 2023 after approval by the ethics committee of the Technische Universität Dresden (BO-EK-11012020) and provision of written informed consent. Enrollment took place at the University Hospital Dresden, Germany. Eligible patients were ≥ 18 years old and had received their first allo-HCT for a hematologic malignancy. Detailed information regarding material and methods can be viewed as supplement.

## Results

Nineteen patients undergoing endoscopy for suspected GI aGvHD were included (Suppl. Table 1, Suppl. Fig. S1). Histopathology identified 12 with GI aGvHD and 7 without GI involvement.

In blood circulating T cells, 9 genes were differentially expressed between groups, without clear functional clustering (Suppl. Fig. S2A). Gene set enrichment analysis showed negative enrichment of pathways such as graft-versus-host disease, NK cell cytotoxicity, and antigen processing in GI aGvHD patients (Suppl. Fig. S2B).

**Fig. 1:**
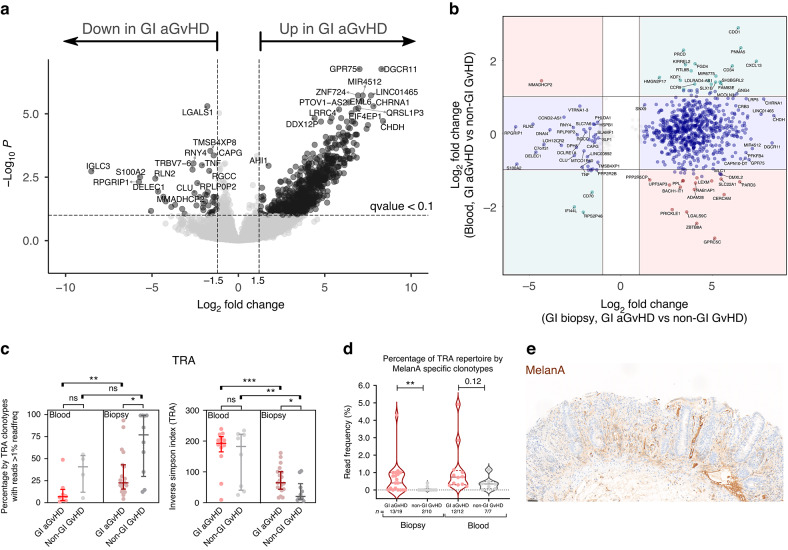
Key gene and TCR signatures revealed by integrated transcriptomic and TCR profiling of gastrointestinal T cells in histopathological confirmed GI aGvHD patients. **a** Gene expression in Biopsy samples: aGvHD vs non-GI aGvHD - Volcano plot of differentially expressed genes in and gastrointestinal T cells at the time of endoscopy of GI aGvHD versus non-GI GvHD patients. Genes with |log2 fold change, log2FC | > 1.5 and q < 0.1 (10% false discovery rate) were considered as significantly differentially expressed. **b** GI aGvHD associated genes from Biopsy in Blood - Categories delineating gene expression patterns of potential GI aGvHD associated genes from gastrointestinal biopsies in peripheral blood samples, along with genes falling into each category. The x-axis represents the log2 fold change in GI aGvHD gastrointestinal T cells compared to non-GI GvHD gastrointestinal T cells (−8 to 8, left to right), while the y-axis indicates the log2 fold change in blood-circulating T cells (−5 to 5, bottom to top). Genes with |log2 fold change, log2FC | > 1.5 in both gastrointestinal and blood-circulating T cells and q < 0.1 in gastrointestinal T cells are included. **c** TRA repertoire diversity in Biopsy and Blood: aGvHD vs non-GI aGvHD - Bulk TCR sequencing of sorted CD3 + T cells showing percentage of reads from clonotypes with read frequency > 1% (y-axis) and inverse Simpson Diversity index (y-axis) for TRA chain. Comparisons were done between GI aGvHD (red) and non-GI GvHD (gray) patients across gastrointestinal biopsies (Biopsy) and corresponding blood samples (Blood). Significant differences are denoted by * (0.01–0.05), ** (0.001–0.01), *** (0.001–0.0001), and **** (< 0.0001) using Mann-Whitney U tests. The median with interquartile range is indicated for each. **d** Percentage of TRA repertoire occupied by MelanA specific clonotypes in Biopsy and Blood: aGvHD vs non-GI aGvHD - Violin plots showing percentage of MelanA reactive TRA clonotypes within the total TRA repertoire (y-axis) compared between GI aGvHD (red) and non-GI aGvHD (gray) patients for gastrointestinal biopsies and peripheral blood samples. Significant differences are denoted by * (0.01–0.05), ** (0.001–0.01), *** (0.001 to 0.0001), and **** (< 0.0001) using Mann-Whitney U tests. **e** Aberrant MelanA expression (brown) in gastrointestinal capillary endothelia of GI aGvHD biopsy from Patient Tx009. Immunohistochemical staining with monoclonal mouse anti-human MelanA antibody (Clone A103) on biopsy obtained via colonoscopy (Colo). Scale bar: 50 µm.

In contrast, gastrointestinal T cells showed pronounced changes: 786 genes were differentially expressed (741 up-, 45 downregulated, Fig. 1a). These were not linked to the predefined GvHD pathway but were associated with diverse processes including adhesion, signaling, and pathogen-related pathways (Suppl. Fig. S2C).

Comparison of biopsy and blood data revealed that most transcriptional changes were tissue-specific: 728 genes were altered only in biopsies, while 22 genes were similarly regulated in both compartments, mainly involving cytokine and chemokine signaling (e.g., CCR9, CXCL13, CD70) (Fig. 1b, Suppl. Figs. S2D, S2E).

TCR repertoire analysis showed lower diversity in gastrointestinal tissue compared to blood in both groups (*P* < 0.01). However, GI aGvHD biopsies displayed higher diversity than non-GI GvHD, along with fewer dominant clonotypes (Fig. 1c, Suppl. Fig. S3A).

Analysis of TCR clonotypes identified a large number unique to GI aGvHD (Suppl. Fig. S3B). Among known antigen-specific clonotypes, viral targets predominated, but a subset recognized human antigens (Suppl. Fig. S3D). Notably, MelanA-specific clonotypes were the most abundant among human antigen-specific TCRs, especially in GI aGvHD biopsies (Suppl. Fig. S3E, S3F, S4).

MelanA-specific clonotypes were present in 68% of GI aGvHD biopsies but less frequent in non-GI samples. Their relative abundance was higher in GI aGvHD tissue, while blood levels were similar across groups (Fig. 1d, Suppl. Fig. S3F, S4).

Immunohistochemistry showed heterogeneous MelanA expression in gut tissue (Fig. 1e, Suppl. Fig. S5, Suppl. Table 2). In selected cases, MelanA expression co-occurred with MelanA-specific TCRs, suggesting a potential role in disease, though this pattern was not consistent across all patients (Suppl.Fig. S5, Suppl. Table 2).

## Discussion

GvHD remains a major complication after allo-HCT, with the gastrointestinal tract as a key target organ. While T cells drive disease pathogenesis, the relationship between local and systemic immune responses is incompletely understood. By integrating gene expression profiling and TCR sequencing from matched gastrointestinal biopsies and blood, our study provides a comprehensive view of tissue-specific and systemic immune dynamics in GI aGvHD, supporting the concept of a predominantly localized immune response.

TCR analysis confirmed lower diversity in gastrointestinal tissue compared to blood, consistent with oligoclonal expansion. However, GI aGvHD biopsies showed higher diversity than non-GI GvHD, likely reflecting the complex antigenic environment of the gut and early polyclonal activation prior to clonal contraction [2, 5–9].

Consistent with previous studies, no shared public TCR clonotypes were identified, underscoring the private and heterogeneous nature of the TCR repertoire in GvHD [2, 5–8]. By leveraging public databases, we distinguished viral-specific TCRs from putative alloreactive clones. Among these, TCRs specific for the human antigen MelanA were unexpectedly prominent in GI aGvHD biopsies, suggesting tissue-specific expansion. Although MelanA is not typically expressed in the gut, its presence in selected patients and co-occurrence with MelanA-specific TCRs raises the possibility of autoreactivity contributing to severe disease, though further validation is required [10, 11]. The origin of this MelanA signal remains unclear and may reflect cross‑reactivity or inflammation‑related staining.

Transcriptomic analysis revealed marked differences between gastrointestinal and blood T cells, further supporting tissue-specific immune responses. Despite numerous differentially expressed genes, no dominant pathways emerged. However, enrichment of Wnt-related and cancer-associated pathways, including SRC and TCF7, suggests a potential regulatory axis influencing local T cell responses in the gut [9, 12].

Comparison of tissue and blood identified only a small subset of shared genes, highlighting limited systemic reflection of local inflammation. Among these, CXCL13 and CCR9 emerged as potential biomarkers and therapeutic targets, given their roles in B cell recruitment [13] and gut homing [14].

No major global differences were observed between steroid-sensitive and resistant patients, but subtle transcriptional changes suggested candidates associated with steroid resistance, including NR3C1, SIK1, AGR2, and IL1RL1 (Suppl. Fig. S6).

This study is limited by bulk sequencing, lack of functional validation, small cohort size, and ongoing steroid therapy at the time of sampling. Future studies using single-cell approaches, larger cohorts, and longitudinal designs will be essential to validate antigen specificities and clarify mechanisms.

In summary, our integrated analysis highlights the importance of tissue-specific immune responses in GI aGvHD and identifies MelanA-reactive clonotypes and signaling pathways as potential contributors to disease pathogenesis and targets for future investigation.

## Supplementary information

Main Supplementary File

Supplementary Table S1

Supplementary Table S2

## Data Availability

The original data of this study are available from the corresponding author, Cornelia Link-Rachner, upon request. The supplemental data contains raw genes and TCR counts. Supplementary Table S1 contains raw gene expression counts per sample and log2 fold changes for condition-wise comparisons. Supplementary Table S2 contains TCR counts and read frequency per sample, with each row representing a unique CDR3 amino acid sequence as observed in a specific sample. Identical CDR3 sequences appearing in multiple samples are listed as separate entries. VDJdb-derived annotations, including antigen specificity with gene and epitope information, are included where available.

## References

[1] Carreras E, Dufour C, Mohty M, Kroger N. The EBMT Handbook: Hematopoietic Stem Cell Transplantation and Cellular Therapies. In: Carreras E, Dufour C, Mohty M, Kroger N, editors. The EBMT Handbook: Hematopoietic Stem Cell Transplantation and Cellular Therapies. 7th ed. Cham (CH)2019.

[2] Goel M, Eugster A, Schetelig J, Bonifacio E, Bornhauser M, Link-Rachner CS. Potential of TCR sequencing in graft-versus-host disease. Bone Marrow Transplant. 2023;58:239–46. 36477111 10.1038/s41409-022-01885-2 PMC10005964

[3] Tian G, Li M, Lv G. Analysis of T-Cell Receptor Repertoire in Transplantation: Fingerprint of T Cell-mediated Alloresponse. Front Immunol. 2021;12:778559. 35095851 10.3389/fimmu.2021.778559 PMC8790170

[4] Pogorelyy MV, Shugay M. A Framework for Annotation of Antigen Specificities in High-Throughput T-Cell Repertoire Sequencing Studies. Front Immunol. 2019;10:2159. 31616409 10.3389/fimmu.2019.02159 PMC6775185

[5] DeWolf S, Elhanati Y, Nichols K, Waters NR, Nguyen CL, Slingerland JB, et al. Tissue-specific features of the T cell repertoire after allogeneic hematopoietic cell transplantation in human and mouse. Sci Transl Med. 2023;15:eabq0476. 37494469 10.1126/scitranslmed.abq0476 PMC10758167

[6] Gkazi AS, Margetts BK, Attenborough T, Mhaldien L, Standing JF, Oakes T, et al. Clinical T Cell Receptor Repertoire Deep Sequencing and Analysis: An Application to Monitor Immune Reconstitution Following Cord Blood Transplantation. Front Immunol. 2018;9:2547. 30455696 10.3389/fimmu.2018.02547 PMC6231291

[7] Koyama D, Murata M, Hanajiri R, Akashi T, Okuno S, Kamoshita S, et al. Quantitative Assessment of T Cell Clonotypes in Human Acute Graft-versus-Host Disease Tissues. Biol Blood Marrow Transplant. 2019;25:417–23. 30359734 10.1016/j.bbmt.2018.10.012

[8] Meyer EH, Hsu AR, Liliental J, Lohr A, Florek M, Zehnder JL, et al. A distinct evolution of the T-cell repertoire categorizes treatment refractory gastrointestinal acute graft-versus-host disease. Blood. 2013;121:4955–62. 23652802 10.1182/blood-2013-03-489757 PMC3682344

[9] Sacirbegovic F, Gunther M, Greco A, Zhao D, Wang X, Zhou M, et al. Graft-versus-host disease is locally maintained in target tissues by resident progenitor-like T cells. Immunity. 2023;56:369–85 e6. 36720219 10.1016/j.immuni.2023.01.003 PMC10182785

[10] Dertschnig S, Evans P, Santos ESP, Manzo T, Ferrer IR, Stauss HJ, et al. Graft-versus-host disease reduces lymph node display of tissue-restricted self-antigens and promotes autoimmunity. J Clin Invest. 2020;130:1896–911. 31917684 10.1172/JCI133102 PMC7108931

[11] Przybyla A, Zhang T, Li R, Roen DR, Mackiewicz A, Lehmann PV. Natural T cell autoreactivity to melanoma antigens: clonally expanded melanoma-antigen specific CD8 + memory T cells can be detected in healthy humans. Cancer Immunol Immunother. 2019;68:709–20. 30783693 10.1007/s00262-018-02292-7 PMC11028361

[12] Lee S, Lee K, Bae H, Lee K, Lee J, Ma J, et al. Defining a TCF1-expressing progenitor allogeneic CD8(+) T cell subset in acute graft-versus-host disease. Nat Commun. 2023;14:5869. 37737221 10.1038/s41467-023-41357-9 PMC10516895

[13] Cao YG, Qin XY, Wang N, Jiang EL, Han MZ, Ma YY, et al. B Lymphocyte Chemoattractant (CXCL13) Is an Indicator of Acute Gastrointestinal GVHD in Murine Model. Inflammation. 2017;40:1678–87. 28688097 10.1007/s10753-017-0609-2

[14] Uehara S, Grinberg A, Farber JM, Love PE. A role for CCR9 in T lymphocyte development and migration. J Immunol. 2002;168:2811–9. 11884450 10.4049/jimmunol.168.6.2811

