# Supplementary material for: Integrated transcriptomic and TCR profiling reveals local immune dysregulation of T cells in Gl aGvHD
Source: Bone Marrow Transplant. 2026 Jun 25;61(8):1121–4. doi: 10.1038/s41409-026-02942-w (PMC13437238; doi:10.1038/s41409-026-02942-w)
Supplement: Supplementary file 1 [file 41409_2026_2942_MOESM1_ESM.pdf]

Integrated Transcriptomic and TCR Profiling Reveals Local Immune Dysregulation of T Cells in GI aGvHD

Manisha Goel[1], Anne Eugster[1], Susanne Reinhardt[2], Ulrike Friedrich[2,3,4], Andreas Dahl[2], Linda Wolters[1], Luise Lieth[5], Katja Steinberg-Bains[1], Marc Schmitz[6,7,8], Tilman D. Rachner[9], Anna R. Poetsch[7,10], Desiree Kunadt[5], Raphael Teipel[5], Jan Moritz Middeke[5], Katja Sockel[5], Friedrich Stölzel[5,11], Malte von Bonin[5], Johannes Schetelig[5], Andreas Hartig[12], Martin Bornhäuser[1,5], Ezio Bonifacio[1] and Cornelia S. Link-Rachner*[1,5]

1 Center for Regenerative Therapies Dresden, Faculty of Medicine, Technische Universität Dresden, Dresden, Germany;
2 Dresden-concept Genome Center (DcGC), Center for Molecular and Cellular Bioengineering (CMCB) Technology Platform, Technische Universität Dresden, Dresden, Germany;
3 German Center for Diabetes Research (DZD e.V.), Neuherberg, Germany;
4 Paul Langerhans Institute Dresden of the Helmholtz Center Munich, Faculty of Medicine and Universitätsklinikum Carl Gustav Carus, Technische Universität Dresden, Dresden, Germany;
5 Medizinische Klinik und Poliklinik I, Universitätsklinikum Carl Gustav Carus, Technische Universität Dresden, Dresden, Germany;
6 Institute of Immunology, Medical Faculty Carl Gustav Carus, Technische Universität Dresden, Dresden, Germany;
7 National Center for Tumor Diseases (NCT/UCC) Dresden, a partnership between DKFZ, Faculty of Medicine and Universitätsklinikum Carl Gustav Carus, Technische Universität Dresden, and Helmholtz-Zentrum Dresden-Rossendorf (HZDR), Dresden, Germany;
8 German Cancer Consortium (DKTK), Partner Site Dresden, and German Cancer Research Center (DKFZ), Heidelberg, Germany;
9 Medizinische Klinik und Poliklinik III, Universitätsklinikum Carl Gustav Carus, Technische Universität Dresden, Dresden, Germany;
10 Biomedical Genomics, Biotechnology Center, Center for Molecular and Cellular Bioengineering, Technische Universität Dresden, Dresden, Germany;
11 Division of Stem Cell Transplantation and Cellular Immunotherapies, University Hospital Schleswig-Holstein, Kiel, Kiel University, Kiel, Germany;
12 Institut für Pathologie, Universitätsklinikum Carl Gustav Carus, Technische Universität Dresden, Dresden, Germany

Supplementary Data

Material and methods

Patient recruitment and cohort

Patients were prospectively recruited between 2021 and 2023 after approval by the ethics committee of the Technische Universität Dresden (BO-EK-11012020) and provision of written informed consent. Enrollment took place at the University Hospital Dresden, Germany. Eligible patients were ≥18 years old and had received their first allo-HCT for a hematologic malignancy. Nineteen patients underwent endoscopy for suspected GI aGvHD (Supplementary Table 1, Supplementary Figure 1A). Histopathological evaluation and Lerner grading (Supplementary Table 1) identified 12 patients with GI aGvHD and 7 without (non-GI GvHD). For patients with both upper (esophagogastroduodenoscopy, EGD) and lower (colonoscopy, Colo) gastrointestinal tract aGvHD, biopsies were analyzed together as part of the GI aGvHD group. In one patient (Tx060), the upper gastrointestinal tract was unaffected (EGD, GvHD 0), while the lower gastrointestinal tract showed

GvHD (Colo, Grade 1-2). In this case, the unaffected biopsy was included in the non-GI GvHD cohort, and the affected biopsy was included in the GI aGvHD cohort. Patients with histopathologically confirmed GvHD in the upper and/or lower gastrointestinal tract were classified as GI aGvHD.

All patients except one (Tx065, non-GI GvHD group) were on systemic steroid therapy. The GI aGvHD cohort was subdivided into steroid-sensitive (SS-GI aGvHD, n = 6), and steroid-refractory/resistant (SR-GI aGvHD, n = 6) cases by steroid response around the time of endoscopy. The SR-GI aGvHD was defined by lack of improvement after 7 days of corticosteroids or the need for second-line therapy (ruxolitinib or mesenchymal stromal cells).

Sample collection and isolation of mononuclear cells

Gastrointestinal biopsies (Biopsy, 3-5 pieces) were obtained by colonoscopy (Colo) or esophagogastroduodenoscopy (EGD). Mononuclear cells were isolated by digestion using gentleMACS^{TM} Octo Dissociator (130-096-427, Miltenyi Biotec) with a tumor dissociation kit (130-095-929, Miltenyi Biotec) according to the manufacturer´s protocol as described previously(1).

Peripheral blood (25-40 ml) was collected in lithium-heparin monovettes (02.1065.001, Sarstedt AG & Co. KG) within 48 h of endoscopy. Mononuclear cells were isolated by density centrifugation using Lymphoprep^{TM} (1114544, PROGEN Biotechnik GmbH).

Flow cytometry and T cell sorting

Mononuclear cells from biopsies and blood were stained with the antibody panel consisting of CD45 (560777, BD Biosciences), CD3 (555342, BD Biosciences), 7AAD (559925, BD Biosciences), CD4 (48-0047-42, Thermo Fisher Scientific), and CD8 (561423, BD Biosciences). T cells (CD3+CD4+ or CD3+CD8+) were sorted at ≥98% purity into RLT buffer (from RNeasy Micro Kit) using 100 μm nozzle on BD FACS Aria Fusion/Aria II as described previously(1). Up to 50,000 T cells per sample were collected. Data were acquired with BD FACS Diva v9.0.1 and analyzed using FlowJo software v10.9.0.

RNA and library preparation

RNA was extracted from sorted bulk T cells with the RNeasy Micro Kit (10977-035, Qiagen). Libraries were prepared using Smart-seq2 method with modifications, quality checked on the Fragment Analyzer (Advanced Analytical), and sequenced on Novaseq 6000 (Illumina, S4 flow cell) in 100 bp paired-end XP mode (>35 million reads/sample) as described previously(1).

Transcriptomic analysis

Raw reads underwent quality control with FastQC. Alignment to the human reference genome GRCh38 (Ensembl 104 splice sites) was performed with STAR aligner (v2.7.10b). Gene level counts were obtained using featureCounts (v2.0.1) as described previously(1).

Differential gene expression was assessed with DESeq2 (v1.42.0)(2) after filtering out genes with zero counts in >50% of samples. Volcano plots were generated using the EnhancedVolcano (v1.20.0) R package. Pathway enrichment was performed using clusterProfiler (v4.10.0)(3) with the KEGG database, applying the Benjamini-Hochberg method for multiple testing correction. Immunoglobulin receptor genes were excluded to avoid bias in enrichment analyses.

TCR repertoire analysis

TCR CDR3 sequences were reconstructed using MiXCR v3.0.13(4), with PCR and sequencing error correction enabled. TCR chains having identical CDR3 amino acid sequences were considered one clonotype. Diversity was quantified using the Inverse Simpson Index after down sampling to the smallest sample size, implemented in immunarch v0.9.0 as described previously(1). Statistical comparisons were made with two-tailed Mann-Whitney U tests.

Clonotypes were annotated against the VDJdb database(5) and categorized as “known” if present in the database or “unknown” if absent. Two-sided Fisher’s exact test was applied to contingency tables comparing MelanA reactive TCR-positive and TCR-negative samples. Analyses and plots were generated in GraphPad Prism (v7.3) and R Studio (v4.0.2) using the packages dplyr, ggvenn, and ggplot2.

References

1. Goel M, Steinberg-Bains K, Eugster A, Wehner R, Tunger A, Reinhardt S, et al. Combining T cell receptor sequencing and transcriptomics to characterize tissue-resident T cells from human gut biopsies. Biochem Biophys Res Commun. 2025;749:151353.
2. Love MI, Huber W, Anders S. Moderated estimation of fold change and dispersion for RNA-seq data with DESeq2. Genome Biol. 2014;15(12):550.
3. Wu TZ, Hu EQ, Xu SB, Chen MJ, Guo PF, Dai ZH, et al. clusterProfiler 4.0: A universal enrichment tool for interpreting omics data. Innovation-Amsterdam. 2021;2(3).
4. Bolotin DA, Poslavsky S, Mitrophanov I, Shugay M, Mamedov IZ, Putintseva EV, et al. MiXCR: software for comprehensive adaptive immunity profiling. Nat Methods. 2015;12(5):380-1.
5. Shugay M, Bagaev DV, Zvyagin IV, Vroomans RM, Crawford JC, Dolton G, et al. VDJdb: a curated database of T-cell receptor sequences with known antigen specificity. Nucleic Acids Res. 2018;46(D1):D419-D27.

Supplementary Figures

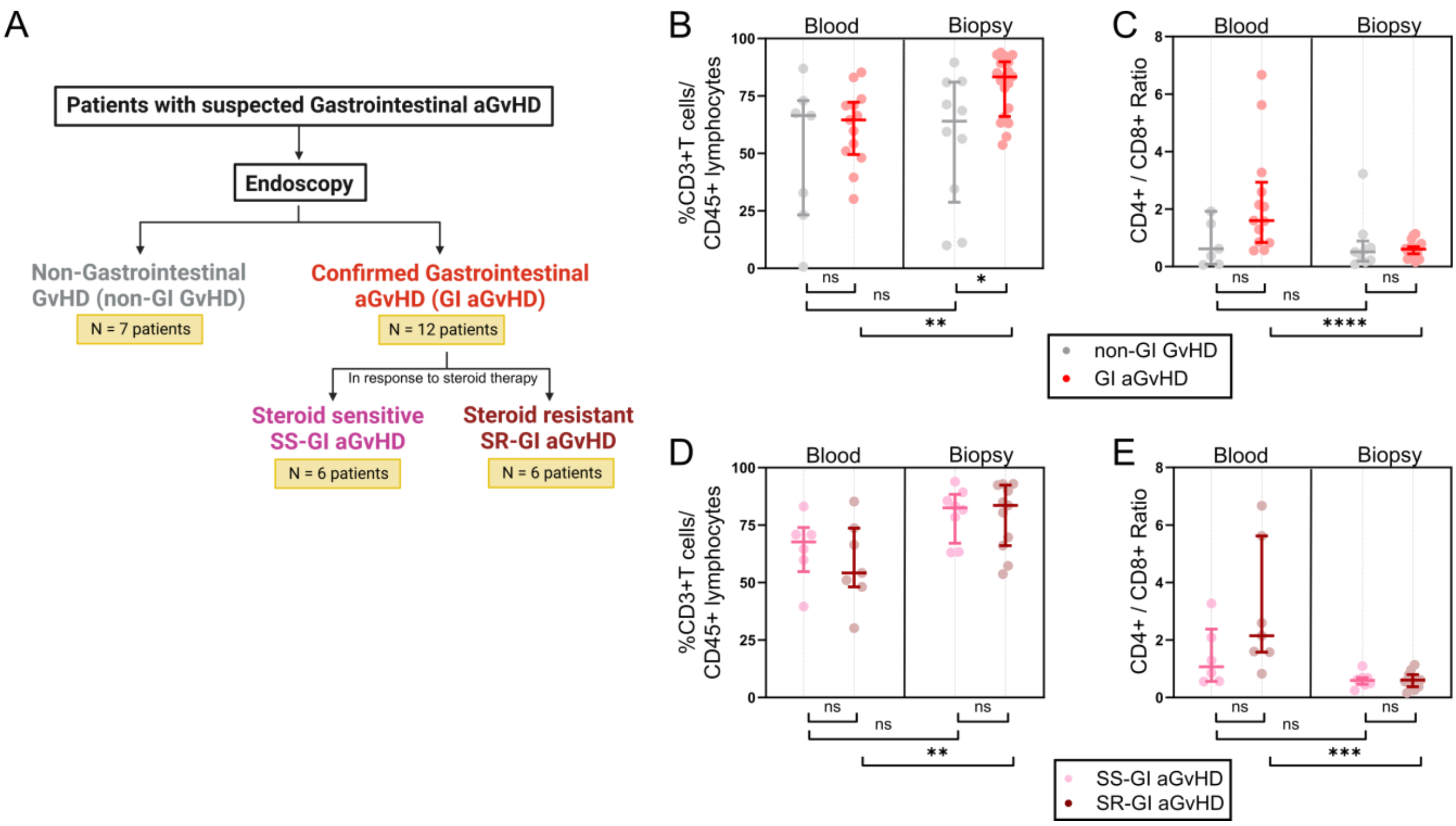

Supplementary Figure S1: Elevated CD3+ T cells observed in gastrointestinal biopsies of histopathological confirmed gastrointestinal acute GvHD patients (GI aGvHD), with an unchanged CD4+/CD8+ ratio, no changes in peripheral sample, and no correlation with steroid resistance. (A) Study design: RNA sequencing of sorted T cells from gastrointestinal biopsies and corresponding blood samples in allo-HCT patients undergoing endoscopy for suspected GI aGvHD. (n = 19; 12 histopathologically confirmed GI aGvHD, and 7 without (non-GI GvHD). The 12 confirmed cases were further categorized based on their response to steroid treatment: 6 responded well (steroid-sensitive, SS-GI aGvHD, pink) and 6 did not respond well, requiring additional treatments (steroid-refractory, SR-GI aGvHD, maroon). **(B, C)** Distribution of T cells in blood samples corresponding to the biopsies obtained within 48 hours of endoscopy and gut biopsies compared between GI aGvHD patients and non-GI GvHD cohort of patients. The percentage of CD3+ T cells out of CD45+ lymphocytes (y-axis, **B**) in biopsies and blood samples, and the CD4/CD8 ratio (y-axis, **C**) in biopsies and blood samples. **(D, E)** Distribution of T cells in

blood samples corresponding to the biopsies obtained within 48 hours of endoscopy and gut biopsies compared between SR-GI aGvHD patients and SS-GI aGvHD cohort of patients. The percentage of CD3+ T cells out of CD45+ lymphocytes (y-axis, **D**) in biopsies and blood samples, and the CD4/CD8 ratio (y-axis, **E**) in biopsies and blood samples. Significant differences are denoted by * (0.01 to 0.05), ** (0.001 to 0.01), *** (0.001 to 0.0001), and **** (< 0.0001) using Mann-Whitney U tests. The median with interquartile range is indicated for each.

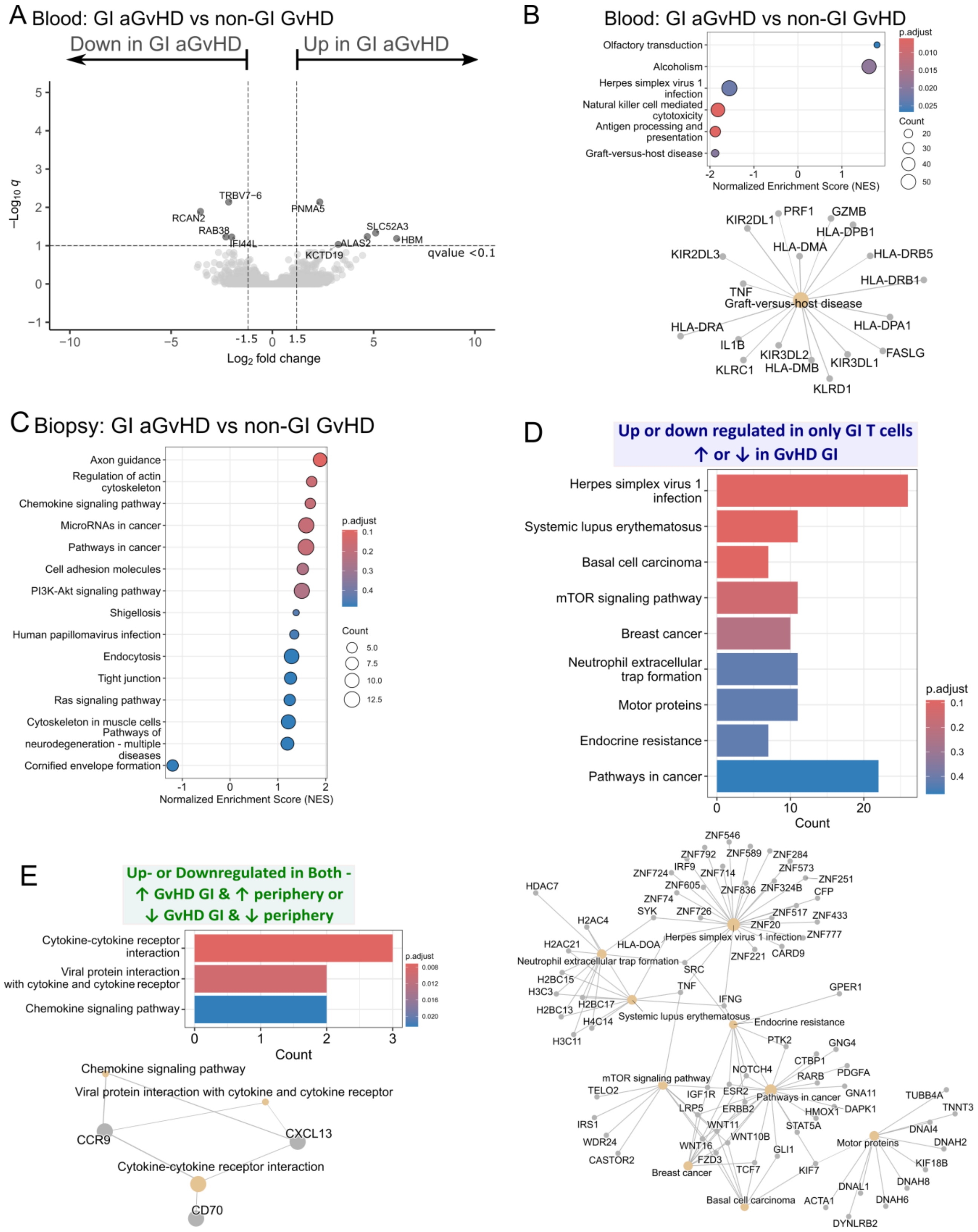

Supplementary Figure S2: CXCL13 (B cell chemoattractant), CCR9 (involved in gut homing), and CD70 (T cell activation regulator) identified as potential GI aGvHD genes in both gastrointestinal and blood-circulating T cells. (A) Volcano plot of differentially expressed genes in blood-circulating T cells at the time of endoscopy of GI aGvHD versus non-GI GvHD patients. Genes with |log2 fold change, log2FC| > 1.5 and q < 0.1 (10% false discovery rate) were considered as significantly differentially expressed. **(B)** KEGG pathways associated with differentially regulated genes in blood-circulating T cells (Top, GSEA Analysis using clusterProfiler, x-axis = Normalized Enrichment Score (NES), y-axis = pathway, circle size = gene count, circle color = significance). Bottom: differentially regulated genes out of all genes associated with “Graft-versus-host disease” pathway. **(C)** KEGG pathways associated with differentially regulated genes in gastrointestinal T cells (GSEA Analysis using clusterProfiler). **(D)** KEGG pathways enriched in genes either upregulated or downregulated exclusively in gastrointestinal T cells of GI aGvHD patients compared to non-GI GvHD patients (ORA using clusterProfiler). Top: enrichment. Bottom: genes contributing to each pathway. **(E)** KEGG pathways enriched in genes either upregulated or downregulated in both gastrointestinal and blood-circulating T cells of GI aGvHD patients compared to non-GI GvHD patients (Over-Representation Analysis, ORA using clusterProfiler). Top: enrichment plot (x-axis = gene count, y-axis = pathway, bar color = significance). Bottom: genes contributing to each pathway.

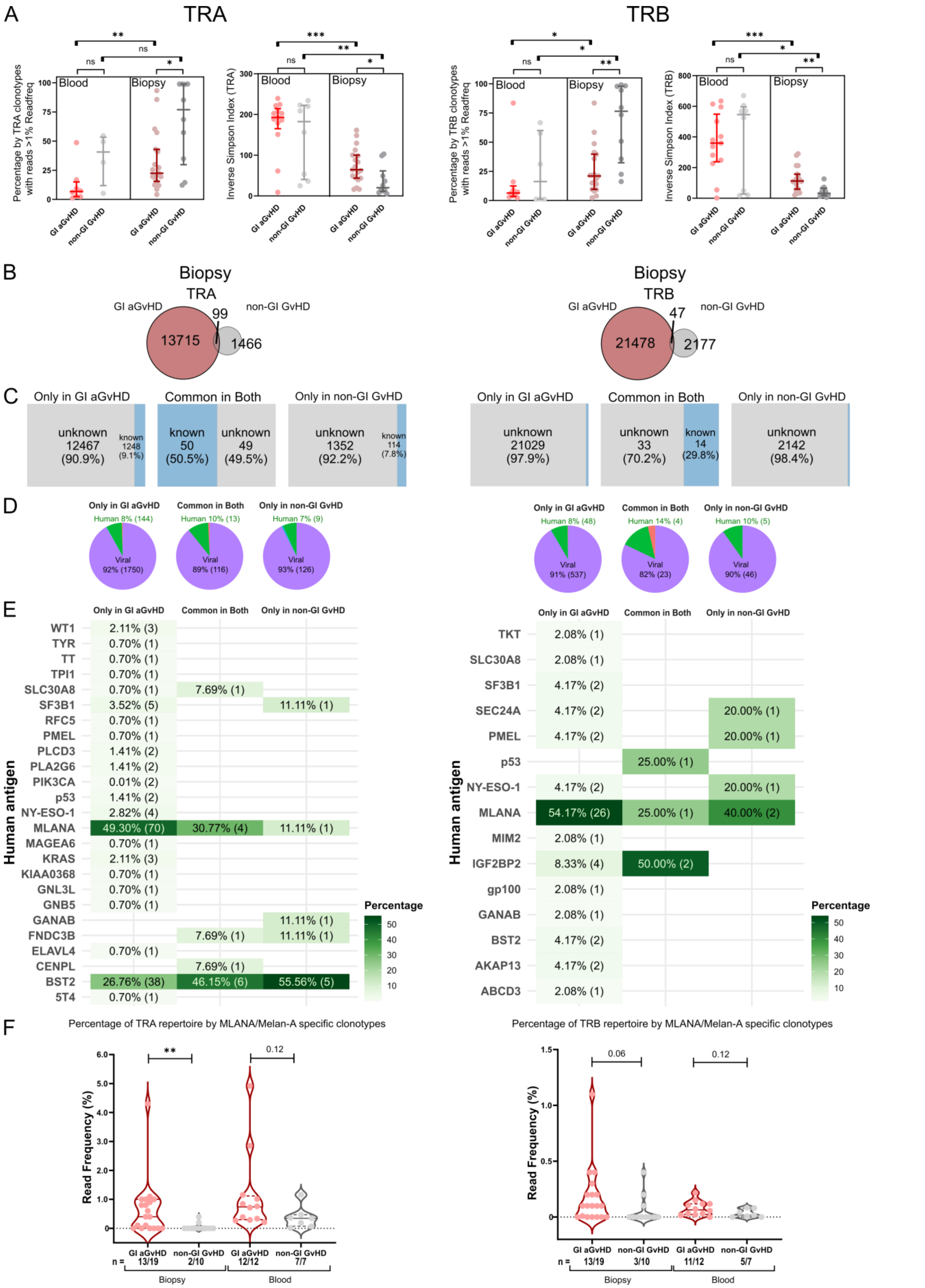

Supplementary Figure S3: GI aGvHD exclusive TCR clonotypes include many known specificities despite a high proportion of unknowns. (A) Bulk TCR sequencing of sorted CD3+ T cells showing percentage of reads from clonotypes with read frequency >1% (y-axis) and inverse

Simpson Diversity index (y-axis) for TRA and TRB chains. Comparisons were done between GI aGvHD (red) and non-GI GvHD (gray) patients across gastrointestinal biopsies (Biopsy) and corresponding blood samples (Blood). Significant differences are denoted by * (0.01 to 0.05), ** (0.001 to 0.01), *** (0.001 to 0.0001), and **** (< 0.0001) using Mann-Whitney U tests. The median with interquartile range is indicated for each. GI aGvHD biopsies showed higher TCR diversity than non-GI GvHD biopsies, though lower diversity in all biopsies compared to blood samples. **(B)** Venn diagram with shared number of unique CDR3 amino acid sequences of TCR clonotypes (TRA, left and TRB, right) between GI aGvHD biopsies (red) and non-GI GvHD biopsies (gray). Based on this, all the identified TCR clonotypes were divided into 3 categories: clonotypes found exclusively in GI aGvHD patients, clonotypes shared between both patient groups, and clonotypes found exclusively in non-GI aGvHD patients. **(C)** Percentage of TCR clonotypes with unknown and known specificities out of all shared and unique TCR clonotypes by screening against the public database VDJdb. **(D)** Percentage of mapped TCR clonotypes with human (green), viral (purple) and other (orange) specificities out of all TCRs with known specificities in each category by screening against the public database VDJdb. **(E)** Heatmap of human antigens (y-axis) corresponding to annotated TCR clonotypes using VDJdb database, showing number and percentage of TCR clonotypes specific to each human antigen found out of total shared (common in both) and unshared (only in GI aGvHD, only in non-GI GvHD) clonotypes from biopsies. (**F**) Violin plots showing percentage of MelanA reactive TCR clonotypes within the total TCR repertoire (y-axis) compared between GI aGvHD (red) and non-GI aGvHD (gray) patients for gastrointestinal biopsies and peripheral blood samples. Significant differences are denoted by * (0.01 to 0.05), ** (0.001 to 0.01), *** (0.001 to 0.0001), and **** (< 0.0001) using Mann-Whitney U tests.

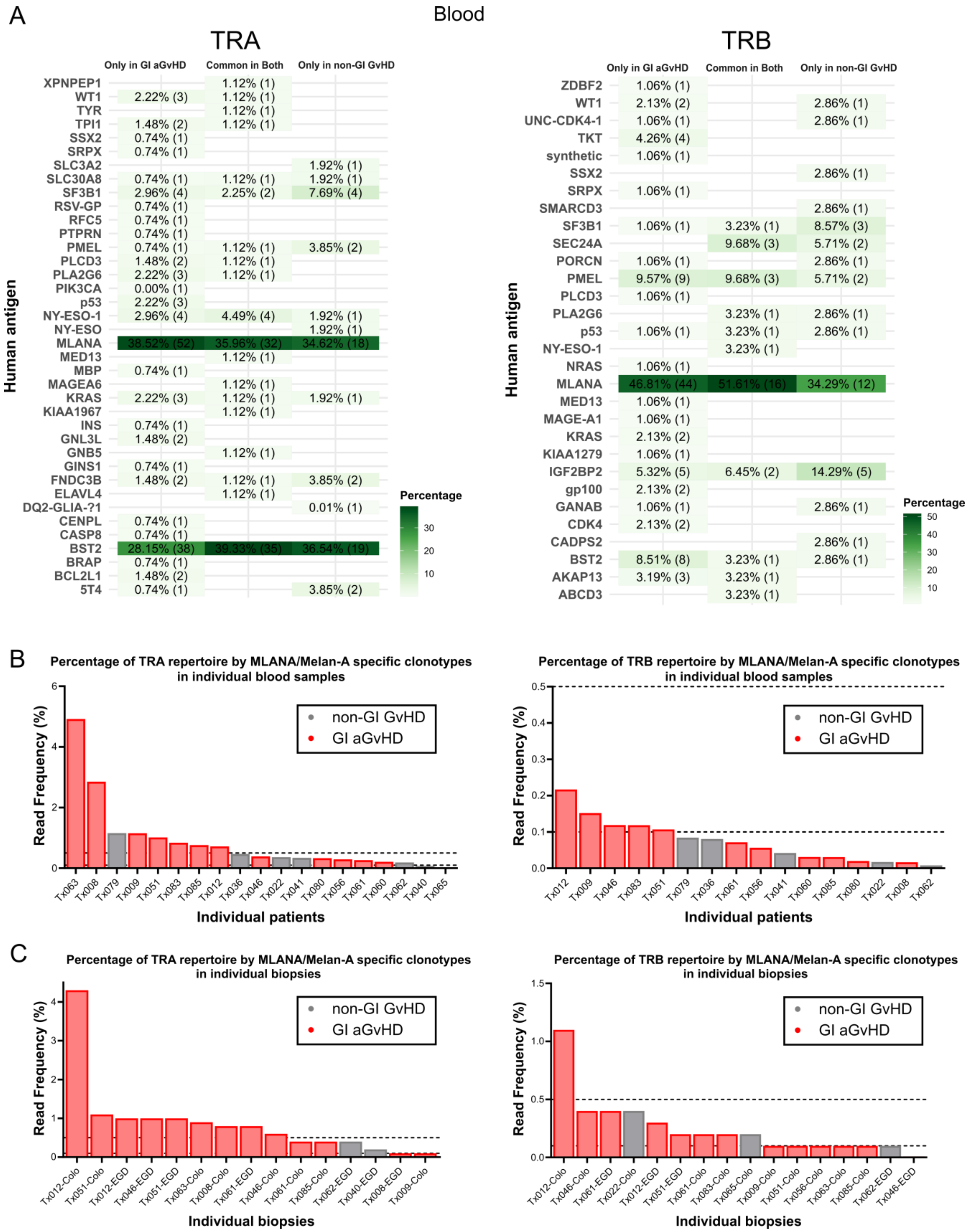

Supplementary Figure S4: MelanA reactive TCR clonotypes in individual blood and tissue samples. (A) Heatmap of human antigens (y-axis) corresponding to TCR clonotypes in peripheral blood samples identified via VDJdb database. The number and percentage of TCR clonotypes specific to each human antigen found out of total shared (common in both) and unshared (only in GI aGvHD, only in non-GI GvHD) clonotypes between GI aGvHD and non-GI GvHD blood samples (x-axis). **(B, C)** Read frequency of MelanA reactive TCR clonotypes out of total TCR repertoire (y-axis)

in individual GI aGvHD (red) and non-GI aGvHD (gray) blood samples **(B)** and gastrointestinal biopsies **(C)**.

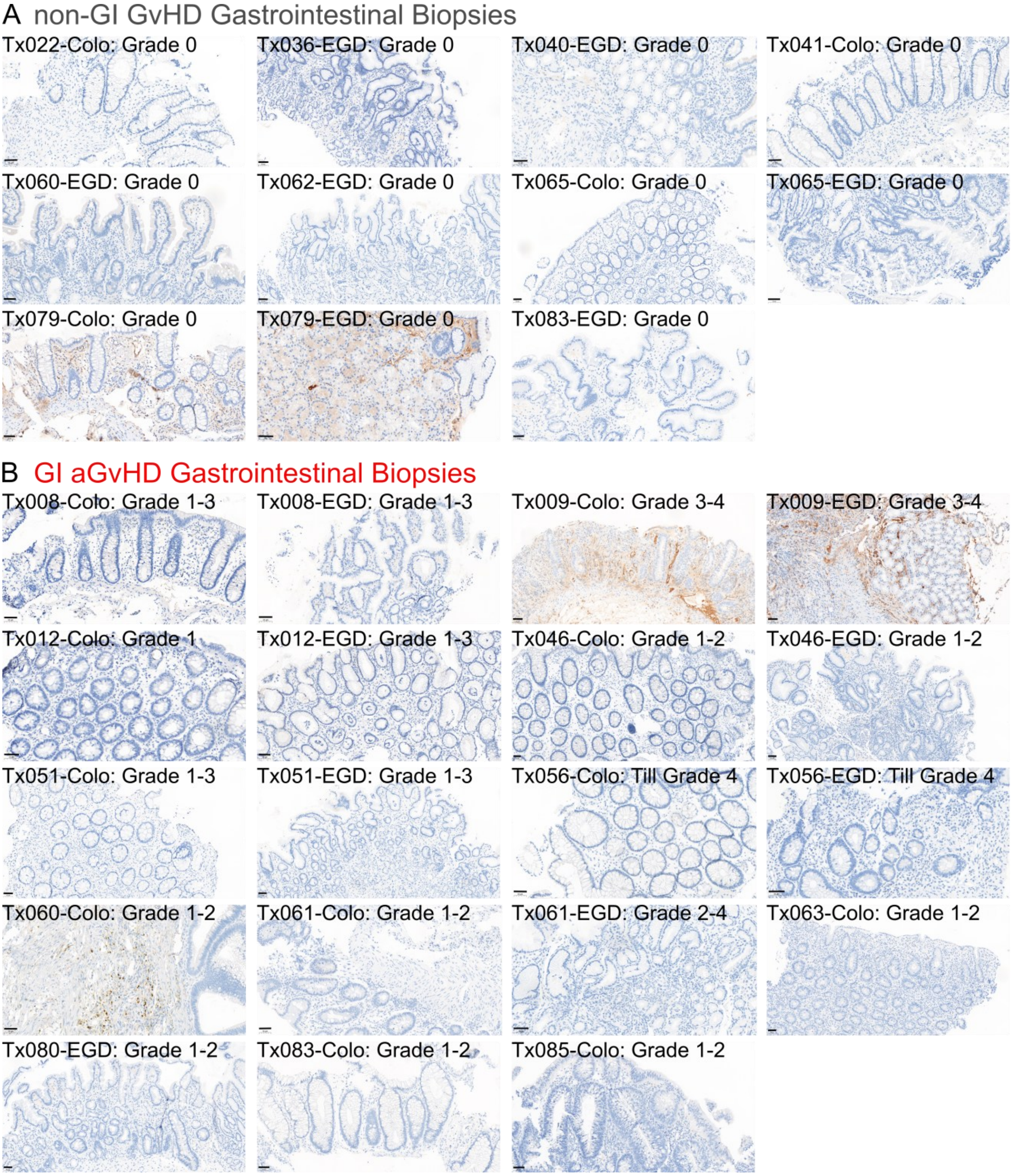

Supplementary Figure S5: Aberrant MelanA/MLANA expression in gastrointestinal capillary endothelia. Immunohistochemical staining with monoclonal mouse anti-human MelanA antibody (Clone A103) on biopsies obtained via colonoscopy (Colo) for the lower gastrointestinal tract or esophagogastroduodenoscopy (EGD) for upper gastrointestinal tract. MelanA, normally absent in gut tissue, was detected in capillary endothelia of two GI aGvHD and one non-GI GvHD patient. **(A)** non-GI GvHD biopsies **(B)** GI aGvHD biopsies, with Patient ID, endoscopy method, and GvHD grading are indicated on each image. Scale bar: 50 µm.

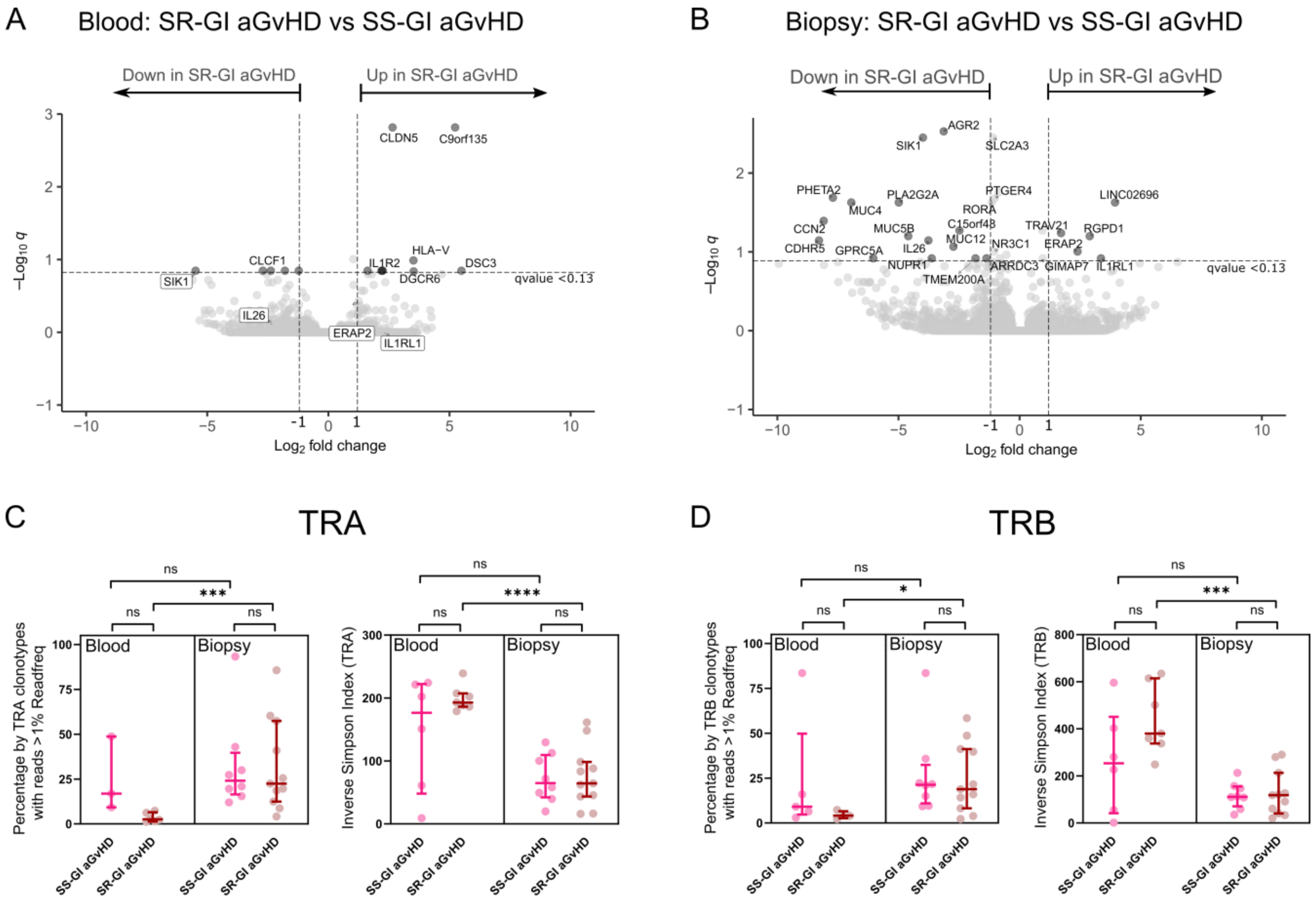

Supplementary Figure S6: Transcriptomic and TCR repertoire differences in steroid resistant (SR-GI aGvHD) versus steroid sensitive (SS-GI aGvHD) patients. (A, B) Volcano plots of differentially expressed genes in blood-circulating T cells **(A)** and gastrointestinal T cells **(B)** at the time of endoscopy in SR-GI aGvHD versus SS-GI aGvHD patients. Genes with |log2FC| > 1 and q < 0.13 (13% false discovery rate) were considered as significantly differentially expressed. Upregulated genes appear on the right, downregulated on the left. The genes significantly differentially regulated in gastrointestinal T cells of SR-GI aGvHD compared to SS-GI aGvHD patients, are also labelled in **A**.

(**C, D**) Bulk TCR sequencing of sorted CD3+ T cells showing percentage of reads from clonotypes with read frequency >1% (y-axis) and inverse Simpson Diversity index (y-axis) for TRA (**C**) and TRB (**D**) chains. Comparisons were done between SR-GI aGvHD (maroon) and SS-GI aGvHD (pink) patients across gastrointestinal biopsies (Biopsy) and corresponding blood samples (Blood). Significant differences are denoted by * (0.01 to 0.05), ** (0.001 to 0.01), *** (0.001 to 0.0001), and **** (< 0.0001) using Mann-Whitney U tests. The median with interquartile range is indicated for each.

Supplementary Tables

Supplementary Table 1. Patients´ characteristics

CLL: Chronic Lymphocytic Leukemia, ALL: Acute Lymphoblastic Leukemia, CMML-2: Chronic Myelomonocytic Leukemia type 2, DLBCL: Diffuse Large B-Cell Lymphoma, AML: Acute Myeloid Leukemia, AML-MR: Acute Myeloid Leukemia with Myelodysplasia-Related changes, HGBL-MY: High-Grade B-Cell Lymphoma with MYC and BCL2 and/or BCL6 rearrangements, MDS-IB1: Myelodysplastic Syndrome with excess blasts type 1, MM: Multiple Myeloma, T-PLL: T-cell Prolymphocytic Leukemia, GI: gastrointestinal, GvHD: graft-versus-host disease, aGvHD: acute graft-versus-host disease, SR: steroid resistance, SS: steroid sensitive, UD-SCT: Unrelated Donor Cell Transplant, MRD-SCT: Matched Related Donor Cell Transplant, Haplo-SCT: Haploidentical Cell Transplant, FLU: Fludarabine, TBI. Total body irradiation, Bu: Busulfan, PTCy: Post-transplant Cyclophosphamide, TREC: Treosulfan, FLAMSA: Fludarabine, Amsacrine and Cytarabine, CsA: Cyclosporine A, MTX: Methotrexate, Tacro: Tacrolimus, MMF: Mycophenolate Mofetil

Patient ID	Diagnosis	Age	Sex	GI aGvHD status	GI aGvHD grade	Day of endoscopy post allo-HCT	Steroid Response	Duration of steroid treatment	Transplant regimen allele matching	Conditioning regimen	Post-grafting Immunosuppression
Tx008	CLL	66	F	GI aGvHD	1-3	d147	SS	d58-d103, d124-129, d143-continuous	UD-SCT 10/10, Permissive mismatch	FLU/TBI 2 Gy	Sirolimus, CsA
Tx009	ALL	60	M	GI aGvHD	3-4	d42	SR	d28-d33, d41-d48	UD-SCT 10/10, Permissive mismatch	FLU/TBI 8 Gy	CsA, MTX
Tx012	CMML-2	60	F	GI aGvHD	1-3	d125, d239	SR	d30-d208, d233-continuous	UD-SCT 10/10	FLU/Bu 4	CsA, MTX
Tx022	DLBCL	40	M	non-GI GvHD	0	d130	-	d45-61, d81-d133	UD-SCT 10/10, Non permissive mismatch	FLU/PTCy	Grafalon, Tacro, MMF
Tx036	AML	52	F	non-GI GvHD	0	d35	-	d28-d61	MRD-SCT 10/10, DPB1 matched	FLU/Bu	CsA, MTX
Tx040	AML-MR	66	F	non-GI GvHD	0	d225	-	d51-d70, d86-d128, d184-continuous	UD-SCT 9/10, Permissive mismatch	FLU/TREO	CsA, MTX
Tx041	HGBL-MYC/BCL2	44	F	non-GI GvHD	0	d28	-	d22-continuous	UD-SCT 10/10, Non permissive mismatch	FLU/Bu2	CsA, MTX
Tx046	AML	64	M	GI aGvHD	1-2	d71	SS	d58-d91	UD-SCT 10/10, Permissive mismatch	FLU/TREO	CsA, MTX
Tx051	AML	25	M	GI aGvHD	1-3	d20	SR	d24-continuous	UD-SCT 10/10, Permissive mismatch	Bu/PTCy	CsA, MTX
Tx056	MDS-IB1	56	F	GI aGvHD	1-4	d80	SR	d24-d39, d68-d225	UD-SCT 10/10, DPB1 matched	FLU/Bu	CsA, MTX
Tx060	AML-MR	67	M	GI aGvHD	0-2	d111	SS	d16-d293	Haplo-SCT	FLU/TREO	CsA, Tacro, MMF
Tx061	MM	37	M	GI aGvHD	1-4	d104	SR	d95-continuous	UD-SCT 9/10, Permissive mismatch	FLU/TREO	Tacro, MTX, Velcade
Tx062	MDS-IB1	55	F	non-GI GvHD	0	d131	-	d55-d102, d117-continuous	MRD-SCT 10/10, DPB1 matched	FLU/Bu	MTX
Tx063	T-PLL	58	F	GI aGvHD	1-2	d46	SS	d45-d91	UD-SCT 10/10, Permissive mismatch	FLU/Bu	CsA, MTX
Tx065	AML-MR	61	F	non-GI GvHD	0	d42	-	not given	UD-SCT 10/10, Permissive mismatch	FLAMSA	PTCy, Tacro, MMF
Tx079	AML	65	F	non-GI GvHD	0	d28	-	d27-81	UD-SCT 10/10, Permissive mismatch	FLU/TREO	Tacro, MMF
Tx080	AML-MR	71	F	GI aGvHD	1-2	d18	SR	d19-d75	Haplo-SCT	FLAMSA	PTCy, Tacro, MMF
Tx083	AML	57	M	GI aGvHD	1-2	d25	SS	s26-d132	UD-SCT 10/10, DPB1 matched	FLAMSA	CsA, Tacro, MMF
Tx085	AML-MR	60	M	GI aGvHD	1-2	d111	SS	d104-d265	UD-SCT 10/10, Permissive mismatch	FLU/TREO	Tacro, MMF

Supplementary Table 2. Detection of MelanA reactive TCR clonotypes and antigen expression across GI aGvHD and non-GI GvHD patients: association with clinical and endoscopic features

Table summarizing Patient ID, type of endoscopy performed (EGD, esophagogastroduodenoscopy or Colo, colonoscopy), GvHD grade, response to steroid treatment, prior history of skin aGvHD, presence of MelanA reactive TCR clonotypes found in the biopsies by TCR sequencing, and MelanA antigen expression detected by immunohistochemistry. MelanA specific TCRs were defined based on sequence similarity to known MelanA reactive clonotypes. SR: Steroid resistant, SS: Steroid sensitive, +: Positive; -: Negative

Patient ID	Endoscopy	GvHD Degree	Steroid response	Prior skin aGvHD	MelanA specific TCR clonotypes	MelanA antigen expression
Tx022	Colo	0	-	-	+	-
Tx036	EGD	0	-	-	-	-
Tx040	EGD	0	-	-	+	-
Tx041	Colo	0	-	+	-	-
Tx060	EGD	0	-	+	-	-
Tx062	EGD	0	-	-	+	-
Tx065	Colo	0	-	-	+	-
Tx065	EGD	0	-	-	-	-
Tx079	Colo	0	-	-	Not available	+
Tx079	EGD	0	-	-	-	+
Tx083	EGD	0	-	-	-	-
Tx008	Colo	1-3	SR	+	+	-
Tx008	EGD	1-3	SR	+	+	-
**Tx009**	**Colo**	**3-4**	**SR**	**+**	**+**	**+**
**Tx009**	**EGD**	**3-4**	**SR**	**+**	**-**	**+**
Tx012	Colo	1	SR	+	+	-
Tx012	EGD	1-3	SR	+	+	-
Tx046	Colo	1-2	SS	+	+	-
Tx046	EGD	1-2	SS	+	+	-
Tx051	Colo	1-3	SR	-	+	-
Tx051	EGD	1-3	SR	-	+	-
Tx056	Colo	Till 4	SR	-	+	-
Tx056	EGD	Till 4	SR	-	-	-
Tx060	Colo	1-2	SS	+	-	+
Tx061	Colo	1-2	SR	-	+	-
Tx061	EGD	2-4	SR	-	+	-
Tx063	Colo	1-2	SS	+	+	-
Tx080	EGD	1-2	SR	+	-	-
Tx083	Colo	1-2	SS	-	+	-
Tx085	Colo	1-2	SS	+	+	-
